# Acute hepatitis and myositis associated with Erythema infectiosum by Parvovirus B19 in an adolescent

**DOI:** 10.1186/1471-2431-14-6

**Published:** 2014-01-13

**Authors:** Maria Koliou, Evaggelia Karaoli, Elpidoforos S Soteriades, Sylvie Pavlides, Stavros Bashiardes, Christina Christodoulou

**Affiliations:** 1 Department of Paediatrics, Archbishop Makarios Hospital, 14 Longou Street, 2027, Strovolos Nicosia, Cyprus; 2 Cyprus Institute of Biomedical Sciences (CIBS), Nicosia, Cyprus; 3 Harvard School of Public Health, Department of Environmental Health, Environmental and Occupational Medicine and Epidemiology (EOME), Boston, MA, USA; 4 Department of Virology, Cyprus Institute of Neurology and Genetics, Nicosia, Cyprus

**Keywords:** Parvovirus B 19, Erythema infectiosum, Hepatitis, Myositis, Cyprus

## Abstract

**Background:**

Erythema infectiosum is the most common clinical manifestation of Parvovirus B19 infection although it has also been associated with rheumatologic diseases and various types of systemic vasculitides. Acute hepatitis and benign myositis however are rarely reported in association with Parvovirus B19 infection.

**Case presentation:**

Here we report a 14-year old male, who developed acute hepatitis and benign myositis associated with erythema infectiosum following Parvovirus B19 infection.

**Conclusion:**

Parvovirus B19 infection has rarely been associated with acute hepatitis and exceptionally rarely with benign myositis. Parvovirus B19 should be considered in the differential diagnosis of acute non-A to E hepatitis and in the case of acute benign myositis presenting with a rash especially in children.

## Background

Parvovirus is a ubiquitous agent commonly infecting children. By 15 years of age, half of children are seropositive to the virus [1]. Parvovirus B19 belongs to the erythrovirus genus, which was named after its pronounced tropism for erythrocyte precursor cells. Erythema infectiosum is the most common clinical manifestation of Parvovirus B19 infection transmitted mainly by respiratory droplets, which often occurs in outbreaks among school-aged children. The illness usually starts with fever and non-specific influenza-like symptoms followed by rash, fever and rheumatic symptoms such as arthralgia or arthritis [2]. In 25% to 50% of cases in immunocompetent patients, infection by Parvovirus B19 is entirely asymptomatic [3]. There are, however, well characterized clinical syndromes associated with infection. Apart from erythema infectiosum, these include non-immune hydrops fetalis and arthropathy [1]. Acute hepatitis has rarely been associated with Parvovirus B19 infection [4-7] and its association with benign myositis is exceptionally rare with only one case having been reported in the literature [8].

In the current case report, we present a 14-year old adolescent who developed acute benign hepatitis and concurrent myositis in the context of erythema infectiosum caused by Parvovirus B19.

## Case presentation

A 14-year old white male, was transferred to our hospital in June 2012 from the Paediatric department of a district hospital because of arthralgias, myalgias and chest pain. The patient was well until 48 hours prior to admission when he started to complain of pain mainly in the upper limbs and chest. The next day he developed fever, rash, malaise and worsening chest pain. A few hours before his visit to the Accident and Emergency department of the district hospital he had a short syncope episode. On the same day he started to feel weakness in both upper and lower limbs, he was unable to walk and was admitted to the district hospital.

The child’s medical history was significant for oral antibiotic therapy for an infected epidydymal cyst. Initially he was prescribed ciprofloxacin for 10 days, then cefuroxime for 7 days and co-trimoxazole for another 7 days. The antibiotic therapy was completed one week before the described health problem. In the two years preceding the episode he had reported two episodes of non-specific allergic rashes.

The laboratory work-up performed on admission at the tertiary referral hospital revealed elevated creatinine phosphokinase (CPK) 7,643 U/L and CK-MB isoenzyme 75.8 U/L. Troponin levels were < 50 mg/dl, alanine aminotransferase 180 U/L, aspartate aminotransferase 253 U/L, γ-GT 72 U/L, alkaline phosphatase 146 U/L, total bilirubin 3.14 mg/dl with direct component 1.04 mg/dl. International normalised ratio (INR) was 1.34, and lactate dehydrogenase 760 U/L.

On the third day of hospitalization the child was transferred to our hospital as a referral and on admission he was relatively well with normal vital signs and temperature 37.6°C. On examination, he had an erythematous rash over the face with relative circumoral pallor. An erythematous maculopapular rash was also present over the entire trunk and the extremities. His muscle strength on the upper and lower limbs was decreased (2/5), and the muscles, especially the calves, were slightly painful on palpation, but there was no swelling. The child was unable to walk even after 6 days of hospitalization. In addition, he was complaining of arthralgias especially of the elbow joints but no findings suggestive of arthritis were detected. No abnormal findings were detected from cardiac, respiratory and abdominal examination.

The rash disappeared on the 7^th^ day leaving the lower limbs with a reticulated or lacelike rash more visible during standing and after bathing. His workup included cardiology evaluation with normal ECG, echocardiogram and serial laboratory tests for re-evaluation of his liver function and CPK (Table 1).

**Table 1:** Laboratory results of the patient during hospitalization and follow up (days counted since admission in the district hospital)

**Laboratory test (normal range)**	**Day 4**	**Day 5**	**Day 6**	**Day 7**	**Day 11**	**One month follow up**
CPK (26 – 171 U/L	7643	5168	2463	325	51	83
CK-MB (0 – 25 U/L)	75.8	44	-	-	-	-
ALT (3 – 41 U/L)	180	161	130	92	78	20
AST (3 – 38 U/L)	253	207	133	59	36	28
INR (0.95 – 1.02)	-	1.30	1.45	1.37	1.02	-
γ-GT (9 – 55 U/L)	72	92	95	101	78	17
T. Bilirubin (0.3 – 1.2 mg/dl)	3.14	3.04	-	-	1.12	1.02
Direct Bilirubin (0.1 – 0.2 mg/dl)	1.04	1.60	-	-	0.30	-
LDH (208 – 480 U/L)	760	700	457	538	433	428
Hb (g/dl)	14.7	13.4	12.9	12.7	13.5	15.3
CRP (neg < 0.5 mg/dl)	9.3	12.0	11.0	9.6	2.0	0.1
ESR (mm)	-	14	16	21	34	9

In summary, the liver enzymes and CPK normalized by day 11. Additional laboratory tests included anti-streptolysin titer 237 IU/ml (normal 0 - 350 IU/ml), negative anti-nuclear antibodies (ANA), negative Mycoplasma IgM, and negative viral studies for IgM antibodies for EBV, CMV, Adenovirus, Measles, and Rubella. Antibodies for hepatitis A, C and Hepatitis B surface antigen were also negative. Serum, lymphocytes and stools were negative for enterovirus RNA and adenovirus DNA as detected by TaqMan based RealTime PCR methods (qualitative assays). Parvovirus B19 DNA as detected by TaqMan based RealTime PCR method [9] was positive in the serum and lymphocytes. No quantitative PCR was done. IgG for Parvovirus B19 were strongly positive, whereas IgM negative.

During hospitalization the patient was treated with penicillin 100,000 IU/kg/24h IV for two days and then switched to cefotaxime 100 mg/kg/24h and azithromycin 500 mg/day to complete a 3-day regimen because of high-grade fever, cough and malaise. Blood culture was negative. On day 8 he became afebrile, while his muscle strength returned to normal levels at day 9, when he was able to walk without pain and at day 11 he was discharged home afebrile and in good condition.

## Conclusions

Erythema infectiosum is the most common clinical manifestation of Parvovirus B19 infection transmitted mainly by respiratory droplets. The illness usually runs in a biphasic course, which starts with fever and non-specific flu-like symptoms [2] followed by rash, fever and rheumatologic manifestations such as arthralgia or arthritis. The second phase develops concurrently with the production of detectable anti-viral antibodies, therefore it is believed that these symptoms are at least partially due to the formation and deposition of immune complexes in the tissues [1]. A temporary suppression of erythropoiesis usually occurs during the first phase of Parvovirus infection, which is however recognizable only in patients with chronic haemolytic disorders [3].

Acute hepatitis has rarely been associated with Parvovirus B19 infection [4-6,10]. In most of these cases the outcome was favourable, although, in one case, hepatitis lasted for up to 9 months [7]. Some studies have implicated Parvovirus B19 in cases of liver failure as a result of fulminant hepatitis; however, other studies did not support this association [11,12]. In our case, hepatitis was rather mild. There was an increase in the transaminase levels and the bilirubin and there was evidence of mild hepatic dysfunction as evidenced by an increase of INR; however, the liver abnormalities self-resolved in a few weeks.

The mechanism by which Parvovirus B19 causes hepatic injury has not been elucidated. Two mechanisms have been proposed: One refers to a direct invasion by the virus that can cause hepatic cell damage [13], while the other involves an indirect action mainly triggered by an immune response against the virus [14]. On the cellular level, globoside, a neutral glycolipid, acts as the main cell receptor for the virus and can be found abundantly on the cell membranes of the erythrocyte and its precursors. This provides a possible explanation for most of the underlying pathology linked to the manifestations of Parvovirus B19 infection including transient aplastic crisis and hydrops fetalis [15]. Globoside has also been detected on the membranes of many other cell types in the human body including the hepatocytes [16]. It seems that all non-erythroid cells are not permissive to the virus, meaning that despite the virus gains entry into the cells, it cannot replicate [3]. However, it has been suggested that despite the virus inability to replicate within a non permissive cell, it retains its ability to produce the non structural protein NS1, which can induce apoptosis of the corresponding infected cell [13,17]. This has also been proposed in the case of the hepatocyte and supports the hypothesis of the direct cytopathic effect of Parvovirus B19 on liver cells [13].

Co-trimoxazole, administered a week prior to illness could in rare instances result in a similar clinical picture. Most side effects are thought to be due to sulfonamide component. A hypersensitivity syndrome can be induced consisting of fever, rashes and organ involvement of varying severity. The rash may be of different types including maculopapular or urticarial type but erythema nodosum, exfoliative dermatitis can also occur usually in association with arthralgias [18]. The liver damage is usually mild, consists of a mild transaminase elevation and resolves within a few weeks. However, in rare cases it may progress to fulminant liver failure [19-21].

In our case, some manifestations such as the liver enzyme increase could partially be attributed to the previous administration of co-trimoxazole. However, the lacy appearance of the rash was very characteristic of parvovirus infection. Furthermore, to our knowledge, myositis was never reported in the literature in association to either trimethoprim or sulfonamides. Eosinophilia, a frequent finding associated with hypersensitivity related manifestations of sulfonamides, was undetected in our case.

In some cases, Parvovirus B19 has been associated with rheumatologic disease in both children and adults [22] and with various types of systemic vasculitis, which include cases of dermatomyositis [7,23]. In our patient, myositis may be differentiated from previously reported dermatomyositis cases mainly because of its very short and benign course and the fact that auto-antibodies such as ANA were all negative. Up to now, about one year after the episode, the patient remains completely healthy. An electromyogram was not performed on the patient because of the rapid improvement of his condition. The very short course and self-resolution of myositis in our patient supports the hypothesis that the underlying mechanism is much more benign and self-limited. The myositis in our patient could probably fit more into the context of acute benign childhood myositis (BACM) as the one encountered at the early convalescent period of several viral infections including influenza [24]. BACM is a very benign condition, which follows a few days after a flu-like illness. The CPK is invariably elevated in all such cases. The calf muscles are mainly affected resulting in weakness and inability to walk. In our case the muscle weakness and elevated CPK was detected during the stage of the rash and arthralgia, which may represent the stage of production of antibodies and the formation of immune complexes [1].

One of the hypotheses in the pathogenesis of BACM associated with influenza virus infection is the direct invasion of the muscle cells by the virus, which however is not permissive and therefore does not allow the virus to replicate within the muscle cells. Therefore the initial infection of the muscle tissue by the virus results in some muscle fibre necrosis sufficient to cause elevation of the CPK but does not cause a frank inflammation of the muscle tissue [25]. However, as in the case of hepatitis, there is still uncertainty as to whether the myositis is caused by the direct invasion of the virus or whether an immune mediated mechanism triggered by the virus leads to muscle injury [26]. Unfortunately, we were unable to clarify this hypothesis since a biopsy was not performed due to the short course of illness.

To our knowledge, there is only one similar case report of benign myositis during infection by Parvovirus B19 [8]. That case developed in the context of Erythema infectiosum caused by Parvovirus B19 in a 9-year old child and was also self resolved a few days later. Our case and the one previously reported add to the scientific knowledge associating Parvovirus B19 with a wide range of diseases in both children and adults. Furthermore, it shows that Parvovirus B19 should be considered in the differential diagnosis of acute non-A to E hepatitis and also in the case of acute benign myositis associated with viral infections presenting with a rash.

### Consent

Written informed consent for publication of this Case report and any accompanying images was obtained from the parent of the patient. A copy of the written consent is available for review by the Editor of this journal.

## Abbreviations

ECG: Electrocardiogram; PCR: Polymerase chain reaction; EBV: Epstein-Barr virus; CMV: Cytomegalovirus.

## Competing interests

The authors declare that they have no competing interests.

## Authors’ contributions

In detail: MK, ES and SB drafted the manuscript. MK, EK conceived of the idea for the study. MK, EK and ESS participated in its design and coordinated and helped to draft the manuscript. SP, SB and CC carried out the viral molecular analysis. SP carried out the immunoassays. All authors contributed to interpretation of the data and the final version of the manuscript. All authors have read and approved of the final version to be published.

## Pre-publication history

The pre-publication history for this paper can be accessed here:

http://www.biomedcentral.com/1471-2431/14/6/prepub
